# Robust nuclear lamina-based cell classification of aging and senescent cells

**DOI:** 10.18632/aging.100414

**Published:** 2011-12-24

**Authors:** Christiaan H. Righolt, Merel L.R. van 't Hoff, Bart J. Vermolen, Ian T. Young, Vered Raz

**Affiliations:** ^1^ Department of Human Genetics, Leiden University Medical Center, Leiden, The Netherlands; ^2^ Quantitative Imaging Group, Faculty of Applied Sciences, Delft University of Technology, Delft, The Netherlands; ^3^ Department of Radiology and Nuclear Medicine, Imaging Division, University Medical Center Utrecht, Utrecht, The Netherlands; ^4^ Manitoba Institute of Cell Biology, University of Manitoba, Cancer Care Manitoba, Winnipeg, Canada

**Keywords:** cell senescence, aging cells, apoptosis, nuclear lamina, image processing

## Abstract

Changes in the shape of the nuclear lamina are exhibited in senescent cells, as well as in cells expressing mutations in lamina genes. To identify cells with defects in the nuclear lamina we developed an imaging method that quantifies the intensity and curvature of the nuclear lamina. We show that this method accurately describes changes in the nuclear lamina. Spatial changes in nuclear lamina coincide with redistribution of lamin A proteins and local reduction in protein mobility in senescent cell. We suggest that local accumulation of lamin A in the nuclear envelope leads to bending of the structure. A quantitative distinction of the nuclear lamina shape in cell populations was found between fresh and senescent cells, and between primary myoblasts from young and old donors. Moreover, with this method mutations in lamina genes were significantly distinct from cells with wild-type genes. We suggest that this method can be applied to identify abnormal cells during aging, in *in vitro* propagation, and in lamina disorders.

## INTRODUCTION

Cellular senescence refers to a decline in cell proliferation, initially described during *in vitro* propagation [1]. *In vivo* senescent cells are suggested to promote biological processes that are associated with aging and cancer progression [2]. Cellular senescence is induced by numerous intra- and extra- cellular stimuli, which lead to changes in many cellular processes. A broad range of molecular markers is used to identify senescent cells [2]. Nevertheless, the identification of senescent cells is insufficient and cell-based methods for identification of those cells are not quantitative [3]. Cellular senescence in marrow stroma cells (MSCs) is associated with spatial changes of the nuclear lamina [4]. Deformed nuclear structure is also exhibited in aging and apoptosis [5, 6], as well as in aging-associated cellular processes like apoptosis [7, 8]. Thus spatial changes of the nuclear lamina could be used to identify aging, apoptotic and senescent cells.

The nuclear lamina is shaped by *lamin A*- and *lamin B*- type genes, and mutations in these genes cause structural deformation of the nuclear envelope. Mutations in *lamin A* cause a broad spectrum of dominant heritable human diseases, collectively referred to as laminophaties [9]. Many of these disorders are aging-associated and, as in aging, are progressive. Identification and quantification of malfunctioning cells could help in diagnosis, monitoring the progression of the disease, and evaluating the effectiveness of therapeutic approaches.

We have developed an image processing method that quantifies the shape of the nuclear lamina from Z-stacks of confocal images. Using three descriptors that directly relate to underlying (bio)physical properties of structure the nuclear shape can be quantitatively described. These objective measures report changes in nuclear shape between healthy and apoptotic cells [10]. Here we demonstrate that changes in nuclear shape during cell senescence and aging are quantifiable and descriptors of the nuclear lamina can be used for robust classification of cell populations. Based on a quantitative description of the nuclear lamina we suggest a model for bending of this structure during cell senescence.

## RESULTS

### Cell senescence can be described by spatial changes in the nuclear lamina

**Figure 1:**
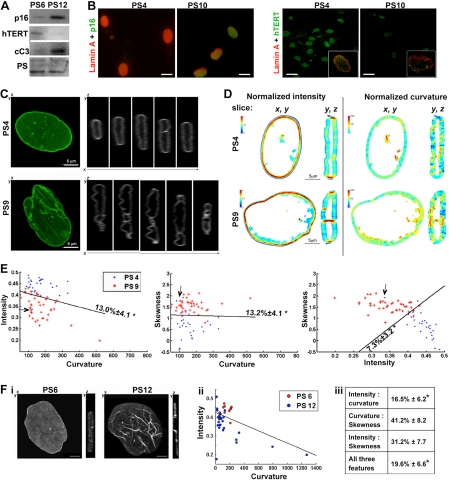
Quantification of nuclear lamina structure changes in senescent cells (**A**) Western blot analysis of protein extracts from hMSCs at passage 6 and 12 (PS6; PS12). p16^INK4a^ (p16) and hTERT marks senescent cells. The cleaved-caspase-3 (cC3) marks apoptotsis. Equal loading control is shown with total proteins staining with PonceauS (PS). (**B**) Maximum projections of confocal images of immunofluorescence of p16^INK4a^ (shown in green; upper row) or hTERT (shown in green; lower row) and lamin A (shown in red) in cultures at passage 4 and passage 10. The box insert shows a single nuclei that was co-stained for hTERT (green) and lamin A (red) is. Scale bars are 10 μm for images with INK4a(p16), and 20 μm for images with hTERT. (**C**) Confocal images of hMSCs expressing WT-lamin A-GFP at passage 4 (PS4), or passage 9 (PS9). Z-stacks were recorded for living cells and 3D-reconstructions were generated. Shown are (*x*,*y*)-plane maximum projection and serial (*y*,*z*)-slices along the x-axis. Scale bar is 5 μm. (**D**) Projections of intensity and curvature values in (*x, y*) and (*y, z*) slice of a typical cell at passage 4 or 9 (PS4, PS9). Left panel shows the intensity, the values are on a linear scale. Right panel shows the curvature, the values are on a logarithmic scale. For each panel images a (*x*,*y*) and a (*x*,*z*) plane are shown. (**E**) Classification of living cells expressing lamin A-GFP at passage 4 (PS4) and passage 9 (PS9). Scatter plots show the distribution of intensity, curvature and skewness in individual cells. A linear classifier is plotted. The classification error and its standard deviation are indicated. An asterisk indicates significant classifications. N_4_ = 28, N_9_ = 40 cells. (**F**) Cell classification between hMSCs at passage 6 and 12 where endogenous lamin A was detected with immunofluorescence. (i) Confocal images of representative nuclei at passage 6 or 12 (ii) Scatter plots show the distribution of intensity and curvature in individual cells. A linear classifier is plotted. (iii) Table shows cross-validations of the classification error ± standard deviation for every two-feature combination and for a test including all three features. Asterisk indicates significant classification. N_6_ = 12, N_12_ = 25, p < 0.01

During *in vitro* propagation, the hMSCs undergo cellular senescence within a few passages and with an associated reduction in cell doubling [11-13]. Cellular senescence of hMSCs is marked by an accumulation of p16^INK4a^ and a decline in hTERT accumulation (Figure 1A). Senescent hMSCs also exhibit changes in the shape of the nuclear lamina [4] and the nuclear lamina is deformed in cells with high p16^INK4a^ expression (Figure 1B). Similarly, misshaped nuclear lamina are exhibited in cells at passage 10 with undetectable hTERT expression (Figure 1B). This cell-based analysis indicates that these changes in lamina shape are associated with cellular senescence. An unbiased description of the lamina shape could help to identify these cells.

Recently we developed a method for quantification of the three-dimensional (3D) structure of the nuclear lamina [10]. This method was successful in discriminating between healthy and apoptotic cells that show massive changes in the nuclear lamina shape [10]. In the current study we have applied this method to fresh and senescent hMSCs, at passage 4 and 9, respectively. The nuclear lamina was visualized with lamin A-GFP, which was expressed with the lentivirus expression system. Z-stacks were taken from confocal images and 3D reconstruction revealed spatial changes in the nuclear lamina during cell senescence (Figure 1C). In fresh cells at passages 4-6, the nuclear lamina structure typically had an elliptical shape, whereas an irregular spatial lamina shape was found in senescent cells between passage 7 and 12 [4]. To examine spatial changes in lamin A the distributions of curvature and intensity in cross sections were compared between fresh and senescent cells. Typical nuclei from each passage are shown in Figure 1D. It is on these types of 3D images that we measure three features: the average normalized intensity (termed *intensity*), the skewness of the intensity distribution (termed *skewness*), and the normalized average absolute Gaussian curvature (termed *curvature*). Formal definitions of these features can be found in [10].

**Figure 2:**
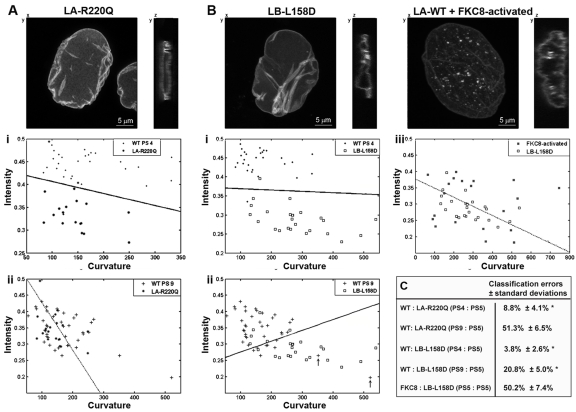
Successful cell classification in cells expressing mutations in lamina proteins Confocal images of a typical cell expressing lamin A (LA)-R220Q-GFP (**A**), lamin B (LB)-L158D-GFP or wild-type lamin A-GFP after FKC8 activation (**B**). Scale bars are 5 μm. Cells expressing WT-LA-GFP are at passage 4 or 9 (PS 4; i), (PS 9; ii) or FKC8-activated (iii). Cells expressing LA-R220Q-GFP, LB L158D-GFP are on passage 5. Scatter plots show the distribution of intensity and curvature in individual cells. Cell numbers are: N_4_ = 28, N_9_ = 40; N_R220Q_ = 21; N_L158D_ = 23 and N_FKC8-activated_ = 20. Arrows indicate cells at passage 9 with high curvature values. A dotted line of the classifier indicates that classification is indistinguishable from a random assignment. (**C**) Table shows cross-validations of the classification error ± standard deviation for a test including curvature, intensity and skewness. Asterisk indicates significant classification, p < 0.01.

Vertical cross sections (*y, z*) revealed substantial changes in the distribution of intensity and curvature values between cells at passage 4 and passage 9 (Figure 1D). High values of normalized intensity were found mainly at the *edges* of the oblate spheroid in fresh cells (Figure 1D, PS4, (*y*, *z*)), but were distributed across the nuclear lamina in senescent cells (Figure 1D, PS 9 (*y*,*z*)). High intensity values of lamin A were also found in intranuclear structures (Figure 2, PS 9 (*y*,*z*)). Typically, intranuclear structures are formed in senescent hMSCs [4]. In most cases, regions with high intensity values of lamin A were surrounded by high curvature values (Figure 1D). Spatial changes of lamin A distribution were less pronounced in horizontal cross sections (Figure 1D; (*x, y*)), suggesting that the structural changes seen in horizontal slices show only part of the spatial changes of the nuclear lamina shape.

Next we investigated whether cell populations at passages 4 and 9 could be distinguished based on a quantitative description of the lamina shape. The values of the intensity, skewness, and curvature were plotted in 2D-scatter plots. Between passages 4 and 9 the distribution of intensity and mean intensity predominantly altered while the curvature changed only slightly (Figure 1E). The 2D-scatter plots revealed clustering of cell populations for any combination of two of the three features (Figure 1E). Classification errors between 7% ± 3.2% to 13% ± 4.1% were found using a linear classifier and cross-validation testing. The p-value of classification errors (*P* < 0.01) indicated that the classification is highly significant in all test combinations. The classification error drops to 6.1% ± 2.9% in a test combining all three descriptors. A few outlier cells were indentified (marked with arrows in Figure 1E). These could represent biological variations. As we aim to develop a method for cell classification that does not require manual manipulation of the dataset outliers were included in all analyses.

To validate the classification results, the lamina quantification method was applied to cell populations at passages 6 and 12 where endogenous lamin A was visualized with immunohistochemistry (Figure 1Fi). After quantification of the nuclear lamina, using the same procedure as described for living cells, a linear classification was found between cells at passage 6 and passage 12 (Figure 1Fii), and this, too, was significant, p < 0.01 (Figure 1Fiii). The classification error in the test for the two features, curvature and intensity, was similar between living and fixed cells, 13.0% ± 4.1% and 16.5% ± 6.2%, respectively. Moreover, in both living and fixed cells the values of mean normalized intensity are reduced in senescent cells. This indicates that changes in lamina structure during cell senescence are not caused by lamin A overexpression, but are induced by senescence.

The analysis of nuclear lamina requires successful segmentation of the 3D nuclear lamina from the rest of the image field-of-view [10]. Two differences were noted between segmentation of living and fixed cells. While 100% of living cells were successfully segmented, in fixed cells only 70% were correctly segmented. Unsuccessful segmentation means that the lamina contour was not completely found and these cells were excluded from further study. In addition, in tests that included the skewness, higher errors were found in fixed cells (Figure 1Fiii). This suggests that during the immunohistochemistry procedure structural artifacts could be generated. In a test including all three descriptors a significant classification was found. Together, following a successful segmentation, lamina-based cell classification between fresh or senescent cells is robust in fixed and in living cells.

### Lamina-based cell classification is highly effective between cell populations

Changes in lamina structure are also induced by mutations in *lamin A* or *lamin B* genes. To evaluate the classification method on cells expressing mutations in a lamina gene, the hMSCs were transduced with a lentivirus expressing lamin A-R220Q-GFP mutant [4] or lamin B-L158D-GFP. Both mutant proteins leads to distorted nuclear shape [8]. Classification tests were performed between cells expressing these mutants at early passage number and a wild type lamin A-GFP expressing cells. At early passage number (passage 5 or 4), cells expressing lamin A-R220Q are significantly distinguished from those expressing wild-type lamin A (Figure 2Ai). Significant classification was not found, however, between cells expressing lamin A-R220Q at passage 5 and cells expressing WT-lamin A-GFP at passage 9 (Figure 2Aii). This is demonstrated by the high classification error >40%, *p* ≈ 0.5. Previously we showed that lamin A-R220Q-GFP expression in hMSCs leads to senescence-associated changes [4]. The lamina classification results suggest that in cells expressing lamin A-R220Q-GFP the nuclear lamina acquires a senescent shape. Cells expressing lamin B-L158D-GFP at passage 5 were significantly distinguished (*p* < 0.01) from young and senescent cells at passage 4 and 9, respectively (Figures 2Bi and 2Bii). Cells expressing lamin B-L158D-GFP show characteristics of apoptotic cells [8], therefore, a comparison to apoptosis-activated cells was preformed. The inducible caspase-8 (FKC8) was expressed in cells expressing lamin A-GFP and 16 hours after FKC8 activation cells were imaged, as previously described in [8]. No classification (*p* ≈ 0.5) was found between cells expressing lamin B-L158D-GFP and those undergoing apoptosis by the activated FKC8 (Figure 2Biii).

**Figure 3:**
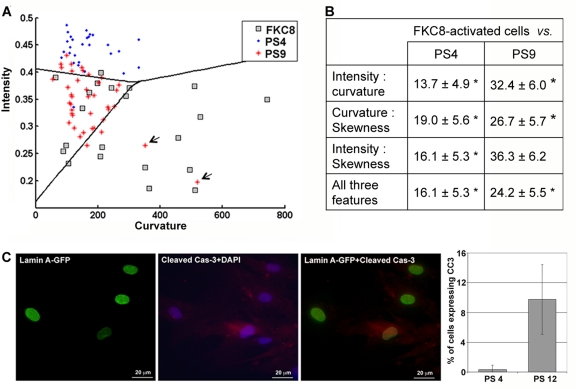
A three-cell population classification assay indicates activation of apoptosis during senescence (**A**) Cell classification between hMSC populations expressing lamin A-GFP at passage 4 (PS4), passage 9 (PS9), and caspase-8-activated cells (FKC8). The scatter plot shows the distribution of intensity and curvature in individual cells at passage 4 (PS4; blue dot) passage 9 (PS9; red cross) or FKC8-activated cells (FKC8; gray square). A linear classification between the three cell populations is indicated. Arrows indicate outliers at passage 9 that have lamina values comparable to apoptotic cells. Cell number per population: N_PS4_ = 28, N_PS9_ = 40 and N_FKC8_ = 25. (**B**) Cross validations of the classification error ± standard deviation are summarized in the table for every feature combination. Asterisk indicates significant classification, p < 0.01. (**C**) Immunolabeling of hMSCs at passage 12with anti-lamin A (green) and anti-cleaved caspase-3 (red) antibodies. Nuclei are counterstained with DAPI. Histograms show the percentage of cells expressing the cleaved-caspase-3 at passage 4 and passage 12. The average and standard deviation over 50 cells is given in the bar plot.

To further evaluate the robustness of the classification procedure, we performed a classification test on three cell populations: passage 4, passage 9 and FKC8-activated cells. The lamina was visualized with lamin A-GFP. A linear classifier was sufficient to distinguish between all three cell populations (Figure 3A). Evaluations of classification errors with a cross-validation test indicated that the classification is highly significant (Figure 3B). Based on the distribution of curvature and intensity values in the cell populations (Figure 3A), we suggest that when cells senesce changes in lamina structure are predominantly associated with a change in intensity, whereas in cells undergoing apoptosis curvature values are also increased.

A few cells at passage 9 (~6%) showed high curvature values similar to these measured in the FKC8-activated cells (Figure 3A, indicated with arrows). This suggests that apoptotic cells could be identified in a senescent cell population. The presence of apoptotic cells was confirmed by cleaved caspase-3 expression in protein extracts of cells at passage 12 (Figure 1A) where ~9% of the cells showed the expression (Figure 3C). Based on the lamina quantification method, 6% of the cells at passage 9 showed curvature values similar to those in FKC8-activated cells. This indicates that activation of apoptosis in senescent cells can be estimated with the lamina quantification method.

### Cell classification between primary myoblasts from young and old donors

**Figure 4:**
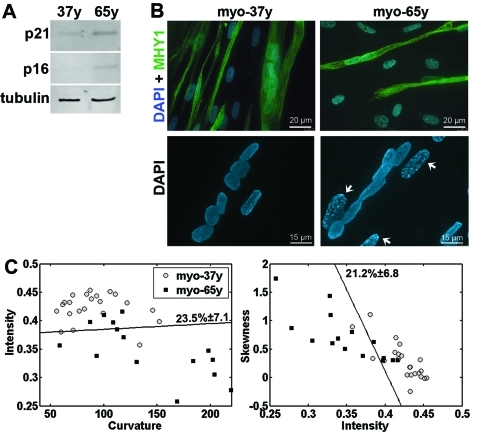
Cell classification in myoblasts from young and old donors (**A**) Western blot analysis of protein extracts from myoblast cultures from donors at 37 and 65 years (37y and 65y) with antibodies to p21^CDKN1A^ and p16^INK4a^. Equal loading control is shown with tubulin. (**B**) Immunohistochemistry with anti-MHY1 antibody in 37y and 65y myotube cultures. Nuclei are counterstained with DAPI. Scale bar is 20 μm. Higher magnification images of DAPI staining show heterochromatic foci (indicated with arrows) in 65y but not in 37y cells. Scale bar is 15 μm. (**C**) 2D scatter plots show the distribution of intensity, curvature, and skewness in individual cells. A linear classifier is plotted. Cross validation of the classification error ± standard deviation are indicated next to the classifier. Both classifications are significant (p < 0.01). N_37y_ = 22, N_65y_ = 15 cells.

In muscle fibers, the shape of the nuclear lamina changes during aging [4]. To evaluate whether the lamina classification method is also suitable to distinguish cells from young and aging individuals, the nuclear lamina shape was examined in primary myoblasts from 37 year-old (myo-37y) and 65 year-old (myo-65y) donors. Fresh myo-65y cells showed key features of senescence including accumulation of p21^CDKN1A^ and p16^INK4a^, heterochromatic foci, and reduced cell fusion, whereas these characteristics were not found in the myo-37y cells (Figures 4A and 4B).

Lamina quantification was performed on endogenous lamin A, which was visualized with immunofluoresence in fresh cultures. Based on the lamina classification method, the myo-37y and myo-65y cell populations were significantly distinguishable (Figure 4C). The intensity values in myo 65y were lower than in the myo-37y cell populations, while skewness and curvature values were higher in the 65y myoblasts, A similar change was found between fresh and senescent hMSCs (Figure 1D). This suggests that structural changes in the nuclear lamina during aging and cellular senescence are similar.

### Local accumulation of lamin A-GFP is associated with reduced protein mobility

**Figure 5:**
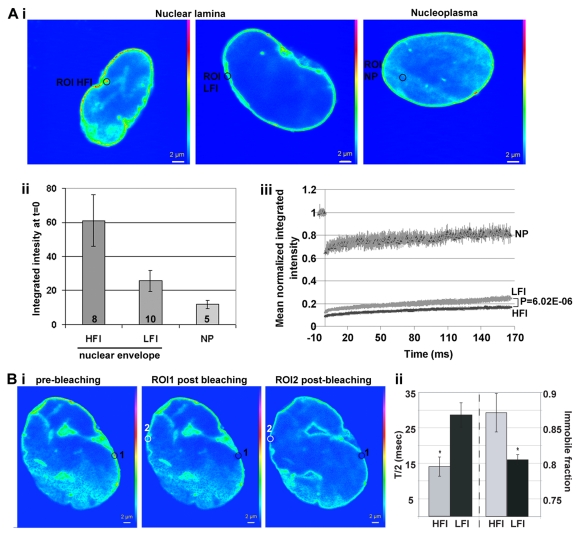
The mobility of lamin A-GFP at the nuclear lamina correlates with protein accumulation FRAP analysis of lamin A-GFP in hMSCs at passage 10. Recovery of fluorescence after photobleaching was performed in selected regions of interest (ROI) with high or low fluorescence intensity (HFI or LFI, respectively). A) shows analysis between cells and B) within cells. FRAP of lamin A-GFP at the nucleoplasm (NP) was used as a control. (**A**) (i) Single confocal sections of representative cells, ROI is indicated with a circle. Scale bar is 2 μm. (ii) Histogram shows the mean integrated intensity in ROI of HFI, LFI or NP prior to bleaching. Number of cells are indicated inside each bar. (iii) Plot shows recovery of lamin A-GFP fluorescence in ROI of HFI, LFI or NP. Averages and SD represent N cells as indicated in (ii). A statistical significant difference of lamin A-GFP mobility between regions with HFI or LFI is demonstrated with P-value < 0.001. (**B**) (i) Single confocal sections of a representative cell (left) before bleaching, (middle) after bleaching of ROI 1 (HFI; indicated with a black circle), and (right) after bleaching of ROI 2 (HFI; indicated with a white circle). Scale bar is 2 μm. (ii) Histogram shows the mean of T/2 and the immobile fraction which was calculated from regions with HFI or LFI. Averages and SD represent 5 cells. Significant difference (*P* < 0.001) T/2 and immobile between HFI and LFI is indicated with an asterisk.

Our studies here revealed that folding of the nuclear lamina is associated with changes in the mean normalized intensity and skewness change during senescence and aging. This suggests that redistribution of lamin A-GFP during cell senescence is associated with local protein accumulation in the nuclear lamina. To investigate this hypothesis, lamin A-GFP mobility was compared between regions with high and low fluorescence intensity (HFI and LFI respectively). Images of representative regions are shown in Figure 5A using the fluorescence recovery after photobleaching procedure (FRAP). As a control, FRAP was carried out on lamin A-GFP in the nucleoplasm (Figure 5A). The mean integrated fluorescence intensity before bleaching in regions with HFI are significantly higher than those of LFI or nucleoplasm regions (Figure 5B). Lamin A in HFI regions is significantly (P < 0.0001) less mobile compared with lamin A in LFI regions (Figure 5B). Confirming previous studies [4], lamin A at the nuclear envelope is far less mobile when compared to lamin A at the nucleoplasm.

To examine differences in lamin A-GFP mobility within a single nucleus, bleaching was carried out on HFI and LFI regions at opposite sides of the nucleus (Figure 5Ci). Statistical analysis of normalized intensity recovery plots revealed that T/2 and the immobile fraction significantly differed between HFI and LFI regions (Figure 5Cii). In HFI regions lamin A-GFP mobility was slower compared to the mobility in LFI regions. Together FRAP results show that local accumulation of lamin A at the nuclear lamina is associated with reduced mobility.

## DISCUSSION

**Figure 6:**
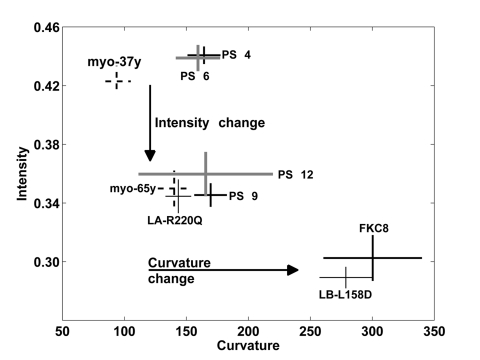
A trend in change of the nuclear lamina between cell populations Plot shows the distribution of the average and standard deviations of the curvature and intensity of the nuclear lamina for hMSCs and myoblasts cell populations. The cell populations at early passage number and myoblasts from a 37 year-old donor (37y) are distinquished from populations of senescent cells, cells expressing lamin A mutant and and myoblasts from a 65 year-old donor (65y), based on average normalized intensity. These cell populations are also separately clustered apoptotic cells based on average curvature.

Mutations in lamin A are known to trigger changes in the nuclear shape [9]. Deformed nuclear shape has also been found in cells undergoing senescence and aging cells [4, 8]. Quantification of the nuclear lamina structure could, therefore, be an objective tool to describe changes in the lamina structure and to identify mutant or unhealthy cells within a cell population. Here we report that based on the nuclear lamina robust and accurate cell classification can be obtained between fresh or young and senescent or aging cells. Statistical analysis of fluorescence intensity and curvature show that cell populations of fresh, senescent and apoptotic cells form separate clusters (Figure 6). Cells expressing mutations in lamina genes also cluster according to their biological definition (Figure 6). Moreover, primary myoblasts from a young and an old donor cluster together with fresh and senescent hMSCs, respectively (Figure 6). This indicates that changes in lamina shape are quantitative, robust and spatial changes of the nuclear lamina between young or fresh cells and aging or senescent cells are similar.

How the shape of the nuclear lamina changes is still an open question. Our analysis shows that between passages 4 and 9 mainly changes in intensity and skewness were found, and at passage 12 values of the curvature also increase. Furthermore, our analysis reveals redistribution of lamin A in the nuclear envelop, which is associated with local accumulation in senescent cells. This suggests that redistribution of lamin A precedes bending. We suggest that protein accumulation above a critical point leads to a structural pressure that causes bending. We have found that local protein accumulation is associated with reduced lamin A mobility in the nuclear envelope. In patients with Hutchinson-Gilford progeria syndrome (HGPS) a point mutation in *lamin A* leads to a deletion of the last axon and the expression of a c-terminus deleted protein [14]. The mobility of progerin is slower compared with the mobility of the mature lamin A protein, which is associated with nuclear distortion [15]. How lamin A mobility at the nuclear lamina is reduced during cell senescence and aging is unclear. The progerin mutation is de novo activated during aging [5], and its expression is increased during *in vitro* propagation [16]. Thus, it is possible that local accumulation of lamin A in senescent cells is caused by aging-associated progerin expression.

Aging is associated with an increase of misfolded proteins and protein aggregation, which in turn is associated with reduced protein mobility [17]. Thus, it is possible that local accumulation of lamin A at the nuclear lamina results from aging-associated protein aggregation. Alternatively, the nuclear shape is affected by the perinuclear actin cap and the LINC complex, which connects the nuclear lamina with the cytoskeleton, is redistributed in cells expressing progerin [18]. Therefore, it is also possible that the uniform distribution of lamin A in the nuclear lamina is disrupted during aging and cell senescence by defects in the LINC complex.

The hMSCs are multipotent and, therefore, an important source for the regeneration of damaged tissues and homeostasis maintenance [19, 20]. The relative ease of hMSC isolation and the potential to differentiate ex-*vivo* into several cell lineages [20] are significant advantages for eventual clinical application. Ex-*vivo*, cell propagation is an essential step before transplantation, but the division rate quickly decreases after 4-6 passages [11, 21, 22]. The method described here could be a relevant tool in the clinic to analyze and sort hMSCs during *in vitro* propagation and prior to transplantation. Efforts to quantify the nuclear lamina contour have been previously reported [23-25]. These studies, however, were not robust and did not describe changes in cellular functionality. The nuclear quantification procedure that we have developed analyzes Z-stack confocal images [10] and is more robust than the other methods that have been reported [23-25]. Moreover, three-dimensional reconstruction of the nuclear lamina, using the descriptive features we have selected, describes the lamina structure in high detail leading to our statistically significant result. We have demonstrated here that this lamina quantification method can be applied on either living cells expressing lamin A-GFP or when lamin A is detected with immunofluorescence. Our lamina-based quantitative imaging method provides a robust and effective method for different cell types and cells expressing mutations in lamina genes. Therefore, it can be applied to identify aging cells and unhealthy cells in lamin A disorders.

## MATERIALS AND METHODS

### Cell culture and lentivirus transduction

The nuclear lamina was visualized with lamin A-GFP, which was expressed with a lentivirus vector previously described in [4, 8]. Activation of FKC8 was carried out with 100 nM AP20187 (ARIAD pharmaceuticals, Cambridge, UK), as described in [8]. Cells were seeded on glass plates prior to imaging. Cells with high lamin A-GFP overexpression, which does not correspond to endogenous lamin A, were excluded.

Human skeletal primary myoblasts from a 37-year-old and a 65-year-old donor (Tebu-bio, Le Perray en Yvelines, France) (named here as 37y and 65y, respectively) were cultured in a medium containing DMEM + 20% Fetal Calf Serum supplemented with an equal volume Skeletal Muscle Cell Media (PromoCell, Heidelberg, Germany) at 37°C under 5% CO_2_. Cells were propagated for only one or two passages and subsequently were seeded on collagen-coated glass plates for imaging.

### Protein detection and analysis

Immunofluorescence and western blotting were carried out as previously described [8]. For protein detection in western blots, aliquots of 50 μg were separated in 12% SDS-PAGE gel. Antibodies in this study are mouse anti-lamin A (Santa Cruz Biotechnology, Santa Cruz, CA, USA) or rabbit anti lamin A (Abcam, Cambridge, UK), rabbit anti-cleaved-caspase-3 (1:200, R&D Systems, Minneapolis, MN, USA), mouse anti-hTERT cloneY182 (Epitomics, Burlingame, CA, USA), mouse anti-human p16 and mouse-anti p21. (Both antibodies were kindly provided by A.G. Jochemsen, Leiden University Medical Center.)

### Image processing and quantification

Cells were recorded using a Leica TCS/SP2 confocal microscope system equipped with a 100x/1.4 NA plan Apo objective. Living cells were imaged at 36.8°C and 5% CO_2_. The spatial sampling distance for the Leica confocal system was Δ*z* = 122 nm/voxel in the axial direction and Δ*x* = Δ*y* = 40 nm/voxel to 60 nm/voxel in the lateral directions. For three-dimensional reconstruction of images, Z-stacks were resampled as described in [25, 26]. The program for identification of the nuclear lamina and quantification of the structure is completely described in [10]. Statistical analysis and the classification tests are performed with PRTools (http://www.prtools.org). The leave-one-out cross-validation of classification error, tests the percentage of N cells that are classified wrongly when one cell is in the test set and N – 1 cells are in the training set. In this way all N cells are tested, one at a time, but at any given stage the cell under tested is not in the training set.

### Fluorescence recovery after photobleaching (FRAP)

FRAP experiments were performed on a Leica TCS/SP5 confocal microscope as described in [17]. Prior to photobleaching, regions were selected based on lamin A-GFP intensity at the nuclear envelope. Fluorescence intensity was determined in single confocal cross sections, using Leica software, version 1.8.0 (Leica Microsystems GmBH, Wetzlar, Germany). Image recording at 0.555-second intervals and 4% laser transmission included 10 and 300 images before or after photobleaching, respectively. The recovery curves were calculated with Excel. Intensity values were corrected for background, fluorescence fading, and photo-bleaching during the image-recording period. P-values were calculated with the T-student test in Excel.
